# Long-term outcomes of the modified Dunn procedure in moderate and severe slipped capital femoral epiphysis: a prospective case series with 7-year follow-up

**DOI:** 10.1186/s10195-026-00899-6

**Published:** 2026-02-10

**Authors:** Mahmoud Fahmy, Ahmed Hazem Abdelazeem, Mostafa Ahmed Shawky

**Affiliations:** Orthopaedic Surgery, Pelvis Fracture and Arthroplasty Unit, Orthopaedic Department, Kasr Alainy Hospital, Cairo University, Cairo, Egypt

**Keywords:** Slipped capital femoral epiphysis, Avascular necrosis, Surgical hip dislocation, Long-term outcomes, Hip preservation surgery, Modified Dunn procedure

## Abstract

**Introduction:**

Slipped capital femoral epiphysis (SCFE) is the most common hip disorder in adolescents and may lead to femoroacetabular impingement, early osteoarthritis, and long-term functional disability if inadequately treated. While in situ pinning remains the standard treatment for mild slips, it fails to correct the deformity in moderate and severe cases, potentially predisposing to degenerative changes. The modified Dunn procedure (MDP) was developed to restore proximal femoral anatomy through surgical hip dislocation while preserving vascular supply.

The aim of the study is to evaluate the long-term radiological and functional outcomes of the MDP in patients with moderate (14 cases) and severe (10 cases) SCFE, and to assess the incidence of avascular necrosis (AVN), osteoarthritis, and other complications.

**Methods:**

A prospective case series was conducted between August 2015 and January 2019 at a single tertiary institution. A total of 24 hips with moderate-to-severe SCFE and open physis were treated using the MDP via surgical hip dislocation. Mild and acute-only slips were excluded. MDP was used as a primary procedure, performed early in severe slips and in selected moderate slips after clinical assessment. Patients were followed clinically and radiologically for a mean duration of 84 ± 2.6 months (range 80–88 months). Functional outcomes were assessed using the Harris Hip Score (HHS) and Merle d’Aubigné and Postel score. Radiographic outcomes and complications, including AVN and secondary arthritis, were documented. Fixation was performed using Schanz screws, cannulated screws, or K-wires according to intraoperative findings.

**Results:**

The mean preoperative slip angle (48.3° ± 7.2°) significantly improved postoperatively (11.4° ± 3.1°, *p* < 0.001). HHS improved from a preoperative mean of 70.4 ± 5.8 to 92.9 ± 4.2 at final follow-up (*p* < 0.001). The Merle d’Aubigné and Postel score improved from 13.8 ± 1.6 preoperatively to 17.5 ± 0.9 at final follow-up (*p* < 0.001). AVN developed in 4 out of 24 hips (16.7%). Arthritis developed in 2 out of 24 hips (8.3%, degenerative OA; no septic arthritis or chondrolysis), representing a total of 6 out of 24 hips (25%) with significant complications when combined with AVN. No cases of postoperative instability or wound infection occurred. Functional scores showed sustained improvement in the majority of patients.

**Conclusions:**

MDP offers favorable long-term anatomical correction and functional recovery in moderate-to-severe SCFE. However, the risk of AVN and arthritis, particularly in unstable or severe cases, warrants careful patient selection and technical precision. Extended follow-up is essential to detect late complications and evaluate procedural durability.

## Introduction

Slipped capital femoral epiphysis (SCFE) is the most frequent adolescent hip disorder, with an incidence ranging from 10 to 15 cases per 100,000 children [1]. It occurs when the proximal femoral epiphysis slips posteriorly and inferiorly relative to the metaphysis through a weakened physis, most commonly during the adolescent growth spurt [2–4]. SCFE can lead to significant long-term morbidity if not treated appropriately, including femoroacetabular impingement (FAI), early osteoarthritis (OA), leg length discrepancy, chronic pain, and loss of hip function [5–7].

Traditional management of SCFE, especially in stable cases, has favored in situ pinning due to its technical simplicity and relatively low risk of avascular necrosis (AVN**) **[8–10]. However, this approach does not correct the underlying deformity of the proximal femur [11–14]. As a result, many patients treated with in situ fixation continue to experience mechanical symptoms and progressive joint damage due to residual cam deformity, particularly in moderate-to-severe slips [15–17]. In situ fixation may also fail to prevent future development of FAI or secondary arthritis, with studies showing high rates of residual deformity and long-term degenerative changes after physeal closure [18–22].

To address these limitations, the modified Dunn procedure (MDP) was introduced, combining surgical hip dislocation with subcapital realignment. This approach allows for direct visualization of the femoral head blood supply, precise correction of the deformity, and preservation of vascular integrity [5–7, 21, 22]. Early results from prospective series have shown promising outcomes in terms of anatomical realignment, restoration of hip biomechanics, and functional recovery [1, 6, 8, 22].

Nevertheless, concerns remain regarding the learning curve, technical complexity, and the potential for complications such as AVN, implant failure, and chondrolysis—especially in unstable slips or when performed by less experienced surgeons [7–9]. Moreover, while many studies report good short-term results, data on the long-term durability of the MDP, particularly in terms of sustained radiographic correction and functional outcomes, remain limited. Most existing literature includes follow-up periods of less than 3 years, often in small, retrospective series. This short follow-up window may underestimate the incidence of late complications such as degenerative arthritis or residual deformities [9–13].

Considering this, the primary aim of our prospective study was to evaluate the long-term radiological and functional outcomes of the modified Dunn procedure (MDP) in patients with moderate-to-severe SCFE. In addition, we aimed to assess the incidence of avascular necrosis (AVN), osteoarthritis, and other complications, including implant-related issues, over a minimum follow-up of 7 years.

## Materials and methods

A prospective case series study was conducted from August 2015 to January 2019 in a university hospital on cases of SCFE. We included patients with open proximal femoral physis who presented with moderate-to-severe SCFE, including both chronic and acute-on-chronic cases, and who were treated with the modified Dunn procedure via surgical hip dislocation. We excluded patients with mild slips or purely acute slips, as these were managed with in situ pinning. Additional exclusion criteria were prior hip surgery, closed physis, or inability to complete the minimum follow-up period of 7 years. Ethical approval was obtained from the institutional ethical committee review board (N-144-205) before data collection. All patients were preoperatively evaluated clinically and radiologically and adequately prepared for the operation. Informed consent was obtained from all individual participants and included in the study. Patient demographics and preoperative data were collected. Standard hip AP and frog lateral radiographs and computed tomography (CT) were obtained. CT was systematically used to quantify slip severity in three dimensions, assess femoral version, and aid surgical planning and correction required. Although CT is not routinely indicated in all SCFE cases, it was considered essential in this series to optimize anatomical correction in moderate and severe slips. Magnetic resonance imaging (MRI) was not included in the preoperative protocol, as the primary focus was on bony morphology and surgical planning rather than early detection of avascular necrosis or intraarticular pathology. Patients were clinically assessed, including stability according to Loder classification [23], chronicity according to Fahey and O’Brien classification [24], degree of slippage on frog lateral X-ray according to Southwick classification [25], and CT, in addition to preoperative functional scores; as well as Harris Hip Score (HHS) [26] and Merle d'Aubigné and Postel score [27].

In this study, MDP was used in all severe and moderate SCFE cases where there was significant slip displacement or risk of progressive deformity. MDP was employed as the primary surgical procedure in all these cases; no patients had undergone prior in situ pinning or other interventions before undergoing MDP. The decision to perform MDP in moderate slips was based on radiographic severity and clinical symptoms, consistent with the selection criteria described in previous studies [1, 2, 4, 5].

All procedures were performed by a dedicated hip preservation orthopedic surgery team with expertise in pediatric and adolescent hip disorders, ensuring optimal management of SCFE and minimizing the risk of complications. Radiographic evaluations and functional assessments were conducted systematically, with a consistent reviewer for all measurements to maintain accuracy and reliability. All cases were done by the same single team under general anesthesia. A dose of prophylactic antibiotic was typically given, usually a first-generation cephalosporin. The modified Dunn procedure was performed according to the Bernese technique [28]. Digastric trochanteric osteotomy is performed. A z-shaped capsulotomy is done to allow for visualization of the epiphyseal slip. In unstable slips, temporary pinning of the femoral epiphysis is done. Viability is ensured by drilling the femoral head. The retinacular soft tissue flaps are developed and mobilized with a periosteal elevator, thus facilitating the development of the posterior retinacular flap without any tension. Eventually, the femoral epiphysis becomes completely mobile but still attached to the posteroinferior retinacular flap. Callus is removed from the metaphyseal stump, and further gradual neck shortening might be needed to allow epiphyseal reduction without tension. Preoperative CT scans were not routinely used to plan the amount of femoral neck resection in three dimensions. Instead, the resection was guided intraoperatively using direct visualization after surgical hip dislocation, combined with fluoroscopic control to achieve anatomic realignment of the epiphysis while preserving the vascular supply. This approach is consistent with most published MDP series, which rely on surgical dislocation technique and careful intraoperative assessment rather than preoperative three-dimensional (3D) imaging [1, 3, 5, 6].

The epiphysis is then reduced manually and fixed preliminarily when a satisfactory alignment is achieved. Vascularity was rechecked and compared to prior testing before reduction, and then the head was relocated. An image intensifier was used to check alignment and to confirm implant position during fixation. Fixation was performed using one of three options on the basis of intraoperative judgment: three 3 mm Schanz screws, or two 6.5 mm fully threaded cannulated screws, or K-wires for capital epiphysis fixation. The fully threaded cannulated screws were placed across the physis to ensure stable fixation. Then, the retinacular flaps and capsule were approximated with loose sutures. The greater trochanter osteotomy fragment was reduced and secured using two 4.5 mm cortical screws. While this may appear to involve multiple fixation points, each implant was applied selectively depending on intraoperative stability and anatomical requirements, ensuring secure fixation while preserving the blood supply to the femoral head [1–6]. Intraoperatively, articular cartilage protection was ensured through careful direct visualization of the femoral head during surgical dislocation, combined with fluoroscopic guidance for precise screw placement. This approach allowed for accurate fixation while avoiding cartilage perforation, without the need for arthrography.

Intraoperative data, including damage to the acetabular or femoral head cartilage and vascularity, as well as postoperative complications were recorded. Postoperative standard hip views were obtained. Active abduction and weight-bearing were restricted until 12 weeks while mobilization in bed was allowed. Patients were followed up at 2 weeks for wound check, then at 6 weeks, 3 months, 6 months, 1 year, and then annually thereafter. Patients were followed up both radiologically and functionally. Radiologically, the postoperative slip angle was recorded and followed up at each visit for any secondary displacement. Appearance of AVN of the femoral head or arthritis were documented at each visit and final follow-up. AVN was diagnosed on plain radiographs and confirmed with MRI. Osteoarthritis was assessed on follow-up radiographs and classified according to the Tönnis classification. All radiographic parameters were evaluated by an experienced hip-preservation orthopedic surgeon, who was blinded to the clinical outcomes. The same evaluator performed all measurements to ensure consistency. Functionally, Merle d'Aubigné and Postel score was documented at 1 year and at final follow-up to be compared with preoperative scores.

### Statistics

Data were coded and entered using the statistical package for the Social Sciences (SPSS) version 26 (IBM Corp., Armonk, NY, USA) and *p* value < 0.05 was regarded as statistical significance. Comparisons between quantitative data were made using unpaired *t*-test and non-parametric Mann–Whitney test. For comparing categorical data, chi-squared (*χ*^2^) test was performed.

## Results

During the study period, 67 cases with SCFE presented to our department, 24 of which met the inclusion criteria. This study included 17 male (70.8%) and 7 female (29.2%) participants with a mean age of 12.96 ± 1.3 years (11–15 years). The study included 10 right hips (41.67%) and 14 left hips (58.33%). In total, 6 cases (25%) were unstable, while 18 cases (75%) were stable, and 15 cases (62.5%) were acute on top of chronic, whereas 9 cases (37.5%) were chronic. Acute slips requiring in situ pinning were excluded from the study; 10 cases (41.67%) were severe slips (Southwick angle more than 50°), while 14 cases (58.33%) were moderate (Southwick angle 30–50°). The mean Southwick angle at presentation was 48.29° ± 8.74° (35–62°). Mean follow-up period was 84 ± 2.61 months (80–88 months) (Table 1).

**Table 1:** Patient demographics and preoperative data (*n* = 24)

Variable	Value
Number of patients	24
Gender	
Male	17 (70.8%)
Female	7 (29.2%)
Mean age (years)	12.96 ± 1.3 (range 11–15)
Side affected	
Right hip	10 (41.67%)
Left hip	14 (58.33%)
Stability (Loder classification)	
Stable	18 (75%)
Unstable	6 (25%)
Chronicity (Fahey classification)	
Acute on chronic	15 (62.5%)
Chronic	9 (37.5%)
Slip severity (Southwick angle)	
Moderate (30–50°)	14 (58.33%)
Severe (> 50°)	10 (41.67%)
Mean Southwick angle at presentation (°)	48.29 ± 8.74 (range 35–62)
Complications	AVN: 4 (16.7%), arthritis: 2 (8.3%), implant migration: 1 (4.16%)
Mean follow-up duration (months)	84 ± 2.61 (range 80–88)

The mean intraoperative blood loss was 559.17 ± 56.41 ml (450–650 ml), and the mean operative time was 119.79 ± 8.78 min (110–140 min). Areas of cartilage delamination and damage of femoral epiphysis were found in two cases (8.3%), which developed arthritis at final follow-up. Intraoperative vascularity test was positive in all cases (100%). None of the stable slips were found to be unstable intraoperatively and vice versa. Fixation was done using three Schanz screws (3 mm) in 8 cases (33.3%), two 6.5 fully threaded cannulated screws in 4 cases (16.7%), or K-wires in 12 cases (50%), with no statistically significant difference as no implant failure or hip instability was observed at final follow-up (Table 2). None of the cases showed secondary displacement or wound infection. One case (4.16%) showed implant migration (using k-wire fixation) into the joint and required surgical removal. This complication highlights a potential risk with this osteosynthesis method, and we recognize that careful consideration should be given when choosing fixation technique.

**Table 2:** Operative details, radiological and functional outcomes, and postoperative complications (*n* = 24)

Parameter	Value
Intraoperative details	
Mean operative time (min)	119.79 ± 8.78 (range 110–140)
Mean intraoperative blood loss (ml)	559.17 ± 56.41 (range 450–650)
Intraoperative vascularity test	Positive in 24 cases (100%)
Intraoperative stability reassessment	No change in stability status for any case
Fixation method	- 3 Schanz screws (3 mm): 8 cases (33.3%) - 2 cannulated screws (6.5 mm): 4 cases (16.7%) - k-Wires: 12 cases (50%)—No implant failure or instability
Cartilage delamination and epiphyseal damage	2 cases (8.3%) → developed arthritis at final follow-up
Wound infection/secondary displacement	None
Radiological outcomes	
Preoperative slip angle (°)	48.29 ± 8.74 (range 35–62)
Postoperative slip angle (°)	11.38 ± 2.79 (range 6–18) *p* < 0.001
AVN development	4 cases (16.7%), mean onset: 8.75 ± 2.5 months
AVN risk factors	- Unstable slips: *p* = 0.0014, severe slips: *p* = 0.041, chronicity: NS (*p* = 0.571)
Arthritis at final follow-up	2 cases (8.3%)
Implant migration	1 case (4.16%) required surgical removal
Functional outcomes (Harris hip score)	
Preoperative HHS	70.42 ± 5.96 (range 60–76)
HHS at 1 year	87.5 ± 4.8 (range 80–96) *p* < 0.001
HHS at 1 year—Grading	- Excellent: 10 cases (41.67%), good: 14 cases (58.33%)
Final follow-up HHS	92.88 ± 7.67 (range 78–100) *p* < 0.001
HHS at final follow-up—Grading	- Excellent: 18 (75%), good: 4 (16.67%), fair: 2 (8.33%)
Functional outcomes (Merle d’Aubigné and Postel score)	
Preoperative score	11.83 ± 1.31 (range 10–14)
Score at 1 year	15.75 ± 1.57 (range 13–18) *p* < 0.001
Grading at 1 year	- Excellent: 2 (8.33%), good: 18 (75%), fair: 4 (16.67%)
Final follow-up score	16.46 ± 2.02 (range 13–18) *p* < 0.001
Grading at final follow-up	- Excellent: 9 (37.5%), good: 9 (37.5%), fair: 6 (25%)
Functional decline (fair scores)	All six cases had AVN or arthritis

Radiologically, the mean slip angle preoperatively was 48.29° ± 8.74° (35–62°), which showed significant improvement postoperatively with a mean of 11.38° ± 2.79° (6–18°), 95% CI 9.8–12.9°; effect size (Cohen’s *d*) = 5.1, *p* < 0.001, highlighting the robustness of MDP. Four cases (16.7%) developed avascular necrosis during follow-up. Cases who were unstable or presented with severe slips showed a statistically significant effect on the development of AVN (*p*-value = 0.0014 and 0.041, respectively). Two cases were detected within the first 6 months postoperatively on MRI, while the other two were identified between 12 and 18 months on routine radiographs. All AVN cases were classified as Ficat stage I–II at diagnosis. Management included protected weight-bearing and close radiological monitoring; none required early surgical intervention. Functionally, these patients showed a decline in both Harris Hip Score and Merle d’Aubigné and Postel scores, contributing to the lower final follow-up scores in this subgroup.

To address differences between moderate and severe slips as well as stable and unstable SCFEs, we performed a stratified analysis. Among the 10 severe slips, 3 cases (30%) developed AVN, whereas only 1 case (7.1%) of the 14 moderate slips developed AVN. Regarding stability, 3 out of 6 unstable slips (50%) developed AVN, compared with 1 out of 18 stable slips (5.6%). Functional scores were also stratified: severe and/or unstable slips showed slightly lower final HHS and Merle d’Aubigné scores compared with moderate and stable slips, largely attributable to cases complicated by AVN or arthritis. As presented in Table 1, this stratification confirms that slip severity and stability are key predictors of AVN and arthritis. However, chronicity or acute on top of chronic presentation had no statistically significant effect on developing AVN (*p*-value = 0.571).

Two hips (8.3%) developed osteoarthritis during follow-up. Both cases were classified as Tönnis grade I–II at the time of diagnosis, representing early degenerative changes. One case corresponded to a hip with prior cartilage delamination observed intraoperatively, while the other developed arthritis gradually over the follow-up period. Neither required surgical intervention at the latest follow-up, but both patients experienced mild functional limitation reflected in slightly lower HHS and Merle d’Aubigné and Postel scores. Overall, the total significant complication rate, combining AVN and progressive OA, was 25%.

Functional scores showed overall improvement at 1 year and at final follow-up as compared with preoperative scores. Preoperatively, HHS had a mean of 70.42 ± 5.96 (60–76) with statistically significant improvement at 1 year with a mean of 87.5 ± 4.8 (80–96) (*p*-value < 0.001). At 1 year, 10 cases (41.67%) had excellent HHS while 14 cases (58.33%) were good. At final follow-up, HHS had shown more statistically significant improvement, with a mean of 92.88 ± 7.67 (78–100) (*p*-value < 0.001). A total of 18 cases (75%) were excellent, 4 cases (16.67%) were good, and 2 cases (8.33%) were fair.

**Fig. 1:**
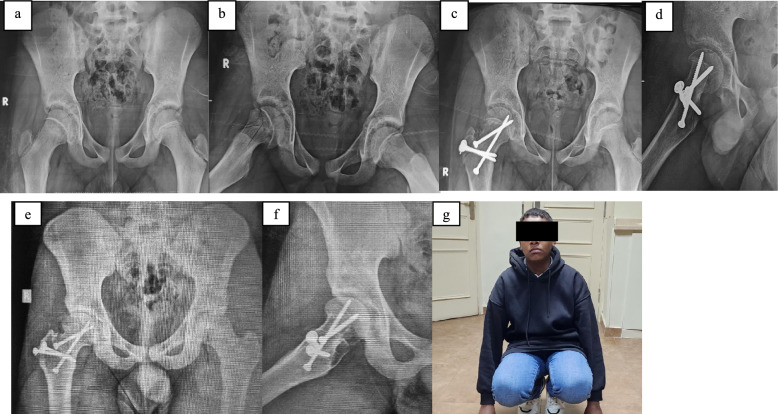
**a**, **b** Preoperative radiographs (ap and lateral) showing right SCFE in 12-year-old boy. **c**, **d** Postoperative radiographs (ap and lateral) follow-up at 1 year follow-up after MDP and fixation by two cannulated fully threaded 4 mm screws. **e**–**g** Postoperative radiographs (ap and lateral) at the final follow-up visit (7 years) and photo showing excellent functional outcome with equal hip flexion range on both sides

**Fig. 2:**
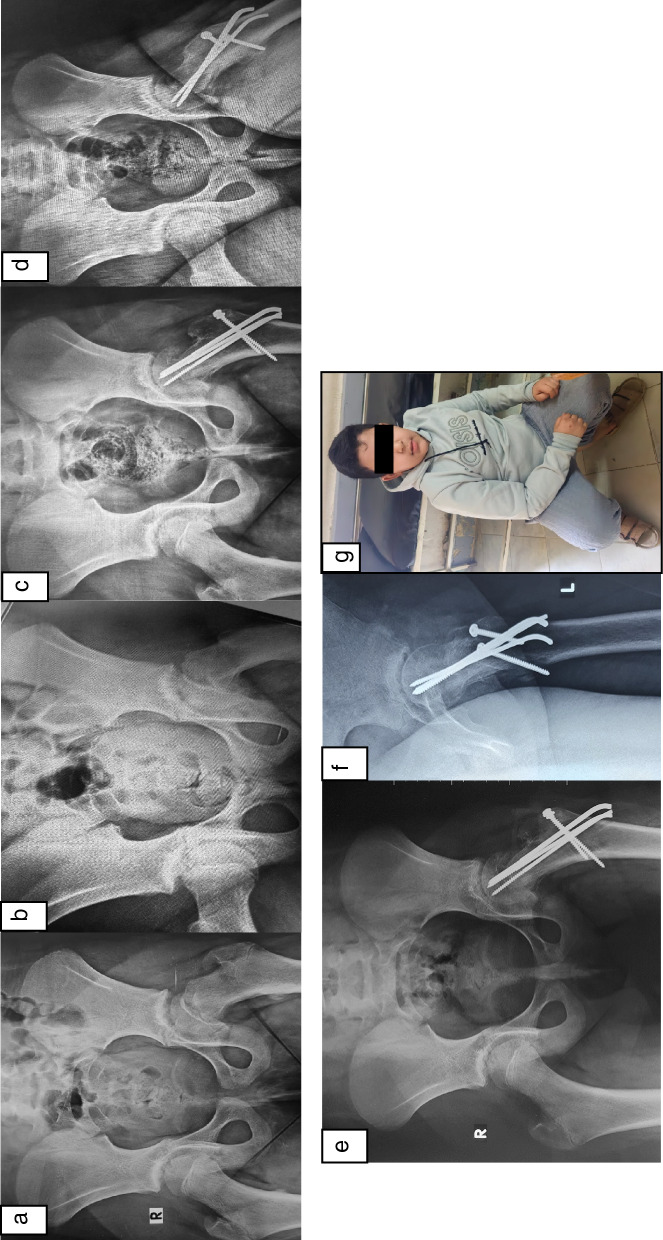
**a**, **b** Preoperative radiographs (ap and lateral) showing left SCFE in 10-year-old boy. **c**, **d** Postoperative radiographs (ap and lateral) follow-up at 1-year follow-up after MDP and fixation by three Schanz screws (3 mm). **e**–**g** Postoperative radiographs (ap and lateral) at the final follow-up visit (7 years) and photo showing excellent normal left hip ROM and function

Merle d'Aubigné and Postel score had a mean of 11.83 ± 1.31 (10–14) preoperatively compared with the score at 1 year, a mean of 15.75 ± 1.57 (13–18) with statistically significant improvement (*p*-value < 0.001). In total, 2 cases were excellent (8.33%), 18 cases were good (75%), and 4 cases (16.67%) were fair. At final follow-up, the mean Merle d'Aubigné and Postel score was 16.46 ± 2.02 (13–18) with overall statistically significant improvement (*p*-value < 0.001). Specifically, all six cases classified as “fair” at final follow-up corresponded to patients who developed either AVN or arthritis, confirming the direct link between complications and poorer functional outcomes. At final follow-up, nine cases (37.5%) were excellent, nine cases (37.5%) were good, and six cases (25%) were fair (Figs. 1 and 2 are case examples).

## Discussion

To our knowledge, our study represents one of the few prospective investigations with such a long follow-up duration, addressing a critical gap in the existing SCFE literature and offering valuable insights for orthopedic surgeons considering anatomical realignment for SCFE treatment. While most existing literature is retrospective and typically limited to mid-term follow-up, our prospective design allows for a more rigorous and reliable assessment of both short- and long-term outcomes (Table 3). This extended follow-up enhances our understanding of the durability of anatomical realignment achieved through surgical hip dislocation, the incidence of complications such as avascular necrosis (AVN), and the long-term functional outcomes. Accurate diagnosis and early recognition of SCFE are critical for selecting the appropriate surgical intervention and optimizing long-term outcomes. Pavone et al. emphasized strategies to avoid diagnostic errors and highlighted key factors that can influence treatment decisions and prognosis. These recommendations reinforce the importance of meticulous preoperative assessment and surgical planning, particularly when considering complex procedures such as the modified Dunn procedure, to minimize complications such as AVN and ensure durable functional results [20].

**Table 3:** Comparison between different studies after MDP for SCFE

Study	Number	Design	Follow-up duration	AVN rate	Main findings/complications
Current study (2025)	24	Prospective	84 months	16.6%	Significant deformity correction; good functional results, no hip instability
Ziebarth et al. (2009) [1]	40	Prospective	24 months	0%	Excellent correction; no AVN or instability
Novais et al. (2015) [2]	48	Comparative retrospective	19.7 months	16%	MDP superior to in situ pinning in correction and function; similar AVN rates
Trisolino et al. (2018) [3]	47	Comparative retrospective	24 months	18.7%	MDP offered better correction; AVN higher in MDP versus pin group
Agashe et al. (2021) [4]	30	Retrospective	24 months	10%	Efficacious in moderate/severe slips; AVN within expected range
Masquijo et al. (2019) [5]	88	Multicenter retrospective	40 months	12.5%	Good radiographic/functional outcomes; some residual deformities noted
Sankar et al. (2013) [6]	36	Retrospective, multicenter	22.3 months	21%	High AVN in unstable slips; technique not protective
Upasani et al. (2014) [7]	67	Retrospective	25 months	14.9%	Learning curve effect; 4% hip instability; complications decreased over time
Novais et al. (2019) [8]	48	Comparative retrospective	19.7 months	16%	MDP superior in correction and function versus pinning; AVN similar
Gabana et al. (2022) [9]	45	Retrospective	28 months	11%	Good correction and AVN within expected range in Brazilian cohort
Nectoux et al. (2015) [10]	222	Retrospective	132 months	–	Not MDP, high FAI and OA rates after in situ fixation
Alves et al. (2012) [11]	20	Prospective	18 months	20%	Surgical dislocation did not reduce AVN in unstable slips
Davis et al. (2019) [12]	36	Retrospective cohort	18 months	16.7%	Similar AVN in stable/unstable SCFE; good function with experienced surgeons
Persinger et al. (2018) [13]	25	Retrospective	20 months	12%	Focused on unstable slips; moderate AVN rate; importance of perfusion monitoring
Abdelazeem et al. (2016) [14]	18	Prospective	18 months	5.6%	Anatomical reduction in stable slips; no hip instability
Birke et al. (2021) [15]	60	Single center retrospective	30 months	9%	Safe in stable slips; no AVN reduction in unstable cases
Sikora-Klak et al. (2019) [16]	48	Retrospective comparative	21.6 months	18.7%	Higher AVN in MDP-treated stable SCFE; triplane osteotomy suggested
Lerch et al. (2019) [17]	40	Retrospective	110 months	5%	Long-term durability showed low OA and AVN; sustained functional results
Aprato et al. (2017) [18]	60	Retrospective	30 months	4%	Hip instability linked to anatomical realignment; emphasized technical precision
Sarassa et al. (2021) [19]	50	Retrospective cohort	30 months	8%	Good early outcomes: long-term follow-up tracked for OA

Our AVN rate reflects the inclusion of high-risk unstable and severe slips and is comparable to those reported in similar populations. Radiological outcomes demonstrated substantial anatomical correction, and functional results were favorable, with most patients regaining near-normal hip function and experiencing minimal pain. The prospective design and extended monitoring period of our study contribute meaningful long-term data to the existing literature, emphasizing the importance of both patient selection and long-term follow-up when evaluating outcomes after the modified Dunn procedure.

In our series, MDP was typically performed as soon as the diagnosis was confirmed and the patient was medically optimized, regardless of chronicity, to achieve anatomical reduction and minimize the risk of further deformity. Consistent with previous studies, early intervention is preferred, particularly for unstable slips, to reduce the risk of progression and subsequent complications [1, 2, 4, 5, 7]. Chronicity, as classified by Fahey and O’Brien, was considered in preoperative planning, but even in chronic cases with significant displacement, MDP was offered when the potential benefits of realignment outweighed the risks of delayed surgery [6, 12]. Our data suggest that earlier intervention after SCFE diagnosis may reduce progressive deformity but does not necessarily eliminate AVN risk, as vascular compromise may already be present in unstable or severe slips [6, 12, 13]. Although our sample size is limited for formal statistical analysis, non-parametric correlation between time from SCFE onset to MDP and AVN occurrence could be explored in larger multicenter datasets to further clarify this relationship.

In our study, fixation during MDP was achieved using a combination of Schanz screws, fully threaded cannulated screws, and k-wires, depending on the intraoperative assessment and epiphyseal stability. This approach is consistent with several reports in the literature. Ziebarth et al. [1] and Trisolino et al. [3] described using multiple fixation methods, including Schanz and cannulated screws, in moderate and severe SCFE cases undergoing MDP. Similarly, Novais et al. [2], Agashe et al. [4], and Masquijo et al. [5] reported favorable outcomes with combinations of cannulated screws and k-wires for both stable and unstable slips. Although the standard in situ fixation often involves a single large-diameter screw crossing the physis, in the context of MDP, multiple fixation methods are frequently employed to maintain reduction and allow early mobilization, particularly in moderate-to-severe slips. Our findings support the idea that this strategy is safe and effective, as no implant failures or hip instabilities were observed at final follow-up.

Several studies have reported outcomes of moderate and severe SCFE managed without MDP, primarily using in situ pinning or conventional osteotomies. For instance, Novais et al. observed that severe stable SCFEs treated with in situ pinning had lower short-term radiographic improvement compared with MDP, although AVN rates were generally lower [2, 8]. Trisolino et al. and Masquijo et al. demonstrated that patients with severe or unstable SCFEs who did not undergo MDP were at risk of residual deformity, impaired hip function, and secondary femoroacetabular impingement [3, 5]. While AVN rates in non-MDP management are typically lower for stable slips, significant residual deformity can occur, especially in severe displacements.

The radiological and functional outcomes following MDP for SCFE across studies report significant improvements, particularly when the procedure is performed by experienced surgeons in appropriately selected cases [13–15]. Superior radiological correction and functional outcomes in moderate-to-severe SCFE compared with traditional methods such as in situ pinning have been observed in cases of surgical expertise, patient selection, and rigorous intraoperative technique. These studies support the growing consensus that anatomical correction via MDP can better restore joint mechanics and delay or prevent degenerative changes [16–19].

The current study confirms that MDP can yield durable, high-quality anatomical and functional results when performed under optimal conditions. In addition, the evidence supports a strong association between the degree of anatomical correction achieved by MDP and subsequent functional recovery [1, 17]. In our series, anatomical realignment was associated with favorable clinical outcomes, including restoration of near-normal hip motion and high HHS.

While high-quality reduction is generally predictive of good function, incomplete correction may impact outcomes. Even small residual deformities can affect functional recovery [4]. However, in unstable slips, Persinger et al. showed that although adequate radiological correction was often done, functional outcomes were more variable, likely due to higher rates of AVN and associated complications [13]. Nectoux et al. evaluated long-term outcomes of in situ fixation and found high rates of femoroacetabular impingement (FAI) due to residual deformity, reinforcing the rationale for anatomical realignment with procedures such as the MDP [10]. Trisolino et al. found that although the MDP group had a higher rate of AVN compared with in situ pinning, they achieved better radiological correction and hip function [3]. This illustrates the balance between anatomical restoration and risk of complications, underscoring the importance of technical precision and postoperative care.

Avascular necrosis (AVN) remains the most devastating complication following MDP. In our series, we observed AVN rate of 16.7% (4 out of 24 hips) over 84-month follow-up, including 6 unstable slips and 10 classified as severe. However, it is important to note that most AVN cases occur within the first postoperative year, and extended follow-up does not significantly change the incidence of AVN. By contrast, the risk of osteoarthritis progression is strongly influenced by the length of follow-up, and our 7-year surveillance allows for more meaningful assessment of degenerative changes. This distinction highlights that the complication profile observed in this study—particularly the low incidence of OA—provides more clinically relevant long-term information than AVN rates alone. Although extended follow-up allowed for the detection of late AVN, the majority of AVN cases occur within the first year, confirming that AVN risk is primarily determined early postoperatively. In contrast, osteoarthritis and other degenerative changes are better assessed with longer-term follow-up, reinforcing the clinical value of our 7-year surveillance.

Sankar et al. reported an AVN rate of 21% in a multicenter retrospective study focusing on unstable slips [6], while Davis et al. found a similar rate of 16.7% in a mixed cohort of stable and unstable cases [12]. Persinger et al. focused solely on unstable SCFE and reported a 12% AVN rate, reinforcing the idea that vascular compromise plays a central role in the pathogenesis of AVN [13]. However, markedly lower AVN rates have been documented in stable slips when the MDP is performed under optimal conditions. Ziebarth et al. reported no AVN cases in a prospective study of mostly stable hips [1]. Similarly, Abdelazeem et al. reported just one AVN case (5.6%) in 18 stable slips managed with anatomical reduction [14]. Birke et al. found a 9% AVN rate in a single-center retrospective series focused on stable slips [15].

**Fig. 3:**
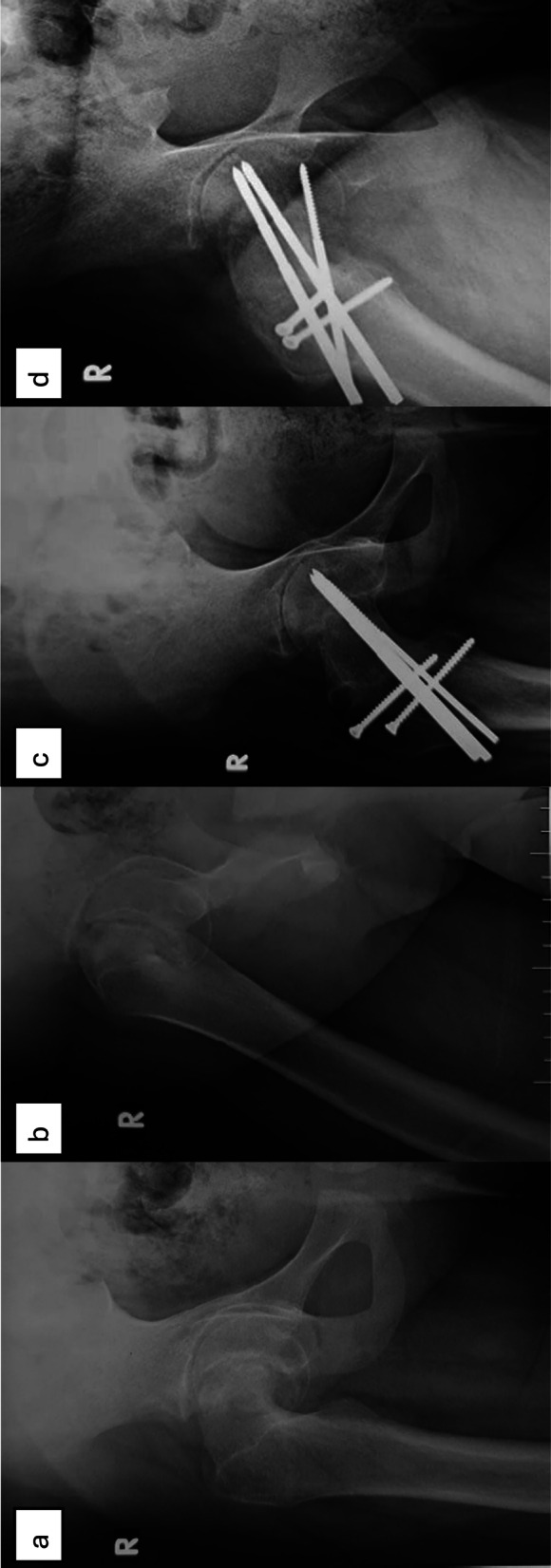
**a**, **b** Preoperative radiographs (ap and lateral) showing right SCFE in 11-year-old girl. **c**, **d** Postoperative radiographs (ap and lateral) at final follow-up visit (7 years) and after fixation by three Schanz screws (3 mm)

**Fig. 4:**
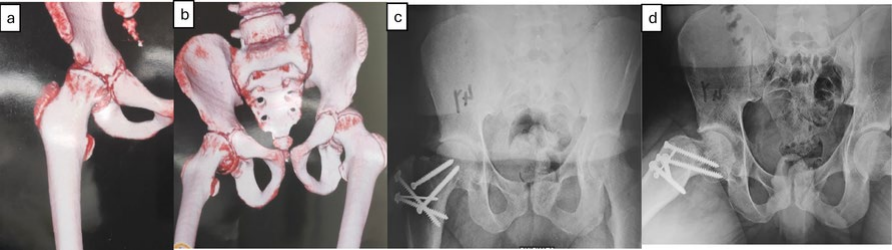
**a**, **b** Preoperative radiographs 3D CT showing right SCFE in 11-year-old boy. **c**, **d** Postoperative radiographs (ap and lateral) at final follow-up visit (7 years) and after fixation by two cannulated screws

**Fig. 5:**
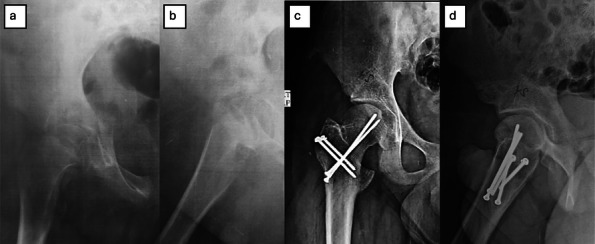
**a**, **b** Preoperative radiographs (ap and lateral) showing right SCFE in 10-year-old boy. **c**, **d** Postoperative radiographs (ap and lateral) at the final follow-up visit (7 year) and after fixation by by two cannulated screws

Slip severity appears to be another independent risk factor for AVN, even in the context of stability. Sikora-Klak et al. reported a high AVN rate (18.7%) in stable, moderate-to-severe slips treated with MDP [16]. Trisolino et al. found the same AVN rate (18.7%) in their MDP group compared with 0% in patients treated with in situ pinning [3], suggesting that the surgical exposure and manipulation required in more severe deformities may increase the risk. Upasani et al., in a study of primarily severe cases, reported an AVN rate of 14.9% [7], while Masquijo et al. reported 12.5% in a multicenter cohort of 88 hips, many of which were severe [5]. Our findings are consistent with this pattern (Figs. 3, 4, and 5).

In our study, there are no cases of postoperative hip instability. Instability rates vary widely across literature depending on patient selection, surgical technique, and experience—being relatively low in experienced hands and particularly in stable SCFE. The current study contributes to this body of evidence even over a long-term follow-up. Alves et al. and Ziebarth et al. did not report instability, but their small sample sizes and limited follow-up reduce generalizability [1, 11]. Birke et al. reported that MDP, when applied to stable SCFE, was not associated with an increased rate of hip instability [15]. Similarly, Masquijo et al. and Sarassa et al. did not report instability as a significant complication, even though their cohorts included both moderate and severe slips [5, 19].

However, some studies draw attention to iatrogenic instability, illustrating the need for precise capsular repair and appropriate re-tensioning of soft tissues during MDP to prevent postoperative subluxation. Upasani et al. reported a 4% incidence of hip instability, attributing it to the steep learning curve associated with MDP [7]. Aprato et al. also emphasized that hip instability could occur if soft-tissue tension is not carefully managed during reduction [18].

Comparison across the reviewed studies shows that long-term complications such as osteoarthritis (OA) and heterotopic ossification (HO) are relatively infrequent after MDP, particularly when performed by experienced surgeons. Lerch et al., with a notably long follow-up, found low rates of osteoarthritis, suggesting the durability of MDP in maintaining joint health over time [17]. Their study supports the idea that anatomical correction may help prevent degenerative changes associated with residual deformity. The current study adds to this evidence, as two cases of osteoarthritis were reported throughout the follow-up period, with no cases of HO. This suggests that, when done correctly and with careful postoperative care, MDP offers a low risk of these degenerative complications in the long term.

However, these complications may occur and vary depending on surgical technique, timing, follow-up duration, and patient-specific factors. Masquijo et al. and Sarassa et al. noted some residual deformities, though the incidence of OA was low during their follow-up periods [5, 19]. This indicates that incomplete correction or less optimal surgical techniques may contribute to later degenerative changes, even with MDP. Nectoux et al. studied patients treated with in situ fixation and reported high long-term rates of FAI and secondary osteoarthritis after 11 years, showing the potential benefit of MDP in avoiding long-term joint degeneration [10].

These data conclude that MDP, when performed with precision and proper technique, can minimize the risk of long-term complications commonly associated with SCFE treatment.

This study is limited by its relatively small sample size and single-center design, which may affect the generalizability of the results. Additionally, the absence of a comparative control group treated by the same surgical team prevents direct comparison with alternative treatments, such as in situ pinning or conventional osteotomies. In addition, the use of different fixation methods introduces heterogeneity and may contribute to variability in outcomes, particularly given the small number of cases in each subgroup. Furthermore, MRI was not included in the preoperative protocol, which may have limited the ability to detect early avascular necrosis or associated intraarticular pathology prior to surgery. Finally, the reliance on plain radiographs limits detailed assessment of femoral head vascularity and early cartilage damage. Future studies with larger, prospective cohorts are needed to evaluate the impact of fixation strategies on clinical outcomes more rigorously. Although MRI was performed in selected cases, the diagnoses of AVN and OA in our study were primarily based on plain radiographs, and no standardized classification systems were consistently applied. This may have reduced sensitivity for detecting subtle early changes and limits direct comparison with other series using uniform MRI-based or validated scoring systems. Future studies using systematic MRI evaluation and standardized classifications would allow for more precise assessment of long-term complications following the modified Dunn procedure.

## Conclusions

The modified Dunn procedure is an effective surgical option for achieving anatomical correction and satisfactory functional outcomes in moderate-to-severe SCFE, including both stable and unstable slips. However, it carries a notable risk of complications, particularly avascular necrosis, hip instability, and long-term degenerative changes. The extended follow-up in this study allowed us to capture late-onset complications, emphasizing the importance of careful patient selection, meticulous surgical technique, and long-term surveillance. These findings reinforce the value of the procedure in complex SCFE cases while highlighting the need for continued refinement of surgical strategies and multicenter prospective studies to further improve outcomes and minimize risks.

## Data Availability

The datasets used and/or analyzed during the current study available from the corresponding author on reasonable request.
